# Overexpression and characterization of two types of nitrile hydratases from *Rhodococcus rhodochrous* J1

**DOI:** 10.1371/journal.pone.0179833

**Published:** 2017-06-23

**Authors:** Yao Lan, Xiaohuan Zhang, Zhongmei Liu, Li Zhou, Ruihua Shen, Xianping Zhong, Wenjing Cui, Zhemin Zhou

**Affiliations:** 1 Key Laboratory of Industrial Biotechnology (Ministry of Education), School of Biotechnology, Jiangnan University, Wuxi, China; 2 ENNova Health Science Technology Co., Ltd., ENN Group, Langfang, Hebei, China; 3 Rudong Nantian Nongke Chemical Engineering Co. Ltd, Yangkou Port Economic Development Zone, Changsha Town, Rudong, China; Tsinghua University, CHINA

## Abstract

Nitrile hydratase (NHase) from *Rhodococcus rhodochrous* J1 is widely used for industrial production of acrylamide and nicotinamide. However, the two types of NHases (L-NHase and H-NHase) from *R*. *rhodochrous* J1 were only slightly expressed in *E*. *coli* by routine methods, which limits the comprehensive and systematic characterization of the enzyme properties. We successfully expressed the two types of recombinant NHases in *E*. *coli* by codon-optimization, engineering of Ribosome Binding Site (RBS) and spacer sequences. The specific activity of the purified L-NHase and H-NHase were 400 U/mg and 234 U/mg, respectively. The molecular mass of L-NHase and H-NHase was identified to be 94 kDa and 504 kDa, respectively, indicating that the quaternary structure of the two types of NHases was the same as those in *R*. *rhodochrous* J1. H-NHase exhibited higher substrate and product tolerance than L-NHase. Moreover, higher activity and shorter culture time were achieved in recombinant *E*. *coli*, and the whole cell catalyst of recombinant *E*. *coli* harboring H-NHase has equivalent efficiency in tolerance to the high-concentration product relative to that in *R*. *rhodochrous* J1. These results indicate that biotransformation of nitrile by *R*. *rhodochrous* J1 represents a potential alternative to NHase-producing *E*. *coli*.

## Introduction

Nitrile hydratase (NHase, EC 4.2.1.84) catalyzes the hydration of a nitrile molecule to the corresponding amide [1]. NHase forms a hetero-tetramer that is composed of α- and β-subunits with either a non-heme iron (Fe-NHase) or non-corrin cobalt ion (Co-NHase) in the active center [2]. The α-subunits, which share a characteristic metal-binding motif [CXLC(SO2H)SC(SOH)] containing two modified cysteine residues, cysteine-sulfinic acid (αCys-SO2H) and cysteine-sulfenic acid (αCys-SOH), bind the metal ions in both Co-NHase and Fe-NHase [1, 3–5]. As observed in previous studies on NHase [6] and a related enzyme, the metal free enzyme is likely to be unmodified thiocyanate hydrolase (SCNase) [7].

The transfer of metal ions into NHases is accomplished by the function-associated protein called the “activator proteins” [8]. The activators for Fe-NHases have been shown to act as metallochaperones [9], whereas the “activator proteins” act as “self-subunit swapping chaperones” for cobalt incorporation into most Co-NHases [8, 10, 11]. Self-subunit swapping is one of the post-translational maturation steps of the cobalt-containing NHase (Co-NHase) family of enzymes [10]. The activator protein exists as a complex with the α-subunit of NHase, and cobalt incorporation into NHase is dependent on the α-subunit swapping between the cobalt-free α-subunit of the cobalt-free NHase and the cobalt-containing α-subunit of the complex [8]. Self-subunit swapping is quite different from the currently known general mechanisms of metal center biosynthesis [8, 10, 12]. Self-subunit swapping was first discovered in the low-molecular-weight NHase (L-NHase) of *Rhodococcus rhodochrous* J1 [8] and, subsequently, in the high-molecular-weight NHase (H-NHase) of the same strain [10]. This swapping is speculated to occur in other Co-NHases and in another NHase family enzyme, thiocyanate hydrolase, which also contains a unique noncorrin cobalt center with two post-translationally modified cysteine ligands [11, 13]. “Self-subunit swapping chaperones” exhibit a surprising protein function in contrast with metallochaperones in metal center biosynthesis and molecular chaperones in protein folding [8, 10].

NHase is widely used for production of acrylamide and nicotinamide in industrial settings. Industrial acrylamide production has been performed with NHase from *Rhodococcus* sp. N774 as the first-generation catalyst and with NHase from *Pseudomonas chlororaphis* B23 as the second generation catalyst [14]. Industrial acrylamide is now produced by NHase from *R*. *rhodochrous* J1 as the third generation catalyst due to its higher catalytic ability and tolerance to acrylamide [14]. The two NHases (H-NHase and L-NHase) from *R*. *rhodochrous* J1 have been expressed in *R*. *rhodochrous* ATCC12674 [15] and in *R*. *facians* DSM43985 [8], respectively, and the two NHases have been well-characterized. Although H-NHase and L-NHase have been successfully expressed in these two expression systems, both of them have the same defect as that in the *R*. *rhodochrous* J1, i.e., long incubation time and unstable expression level. The incubation time for *R*. *rhodochrous* J1 and the two expression systems are at least 72 h [16]. Compared to the expression system of *Rhodococcus*, *E*. *coli* has a particular advantage due to its simple cell culture and molecular biological manipulation. *E*. *coli* is expected to develop a genetically engineered *E*. *coli* for overproduction of NHase from *R*. *rhodochrous* J1. However, both H-NHase and L-NHase have not been overexpressed in the *E*. *coli* expression system, the H-NHase and L-NHase expressed by the *E*. *coli* expression system were only slightly detectable on SDS-PAGE, and the activity was very low [17].

In this study, we successfully expressed the two types of NHases from *R*. *rhodochrous* J1 via codon-optimization, substitution of stronger ribosome binding site (RBS), and optimization of spacing sequences between different genes. Especially, the catalytic properties of H-NHase and the applicable procedure for biotransformation using whole-cell catalysts were systematically determined to provide the basis for industrial transformation. The expressions of the two NHases were stable, and culture times were only 20 h, which was considerably faster than the wild-type strain and the expression system of other *Rhodococcus* (at least 72 h). It is possible that the genetically engineered *E*. *coli* could be an alternative to *R*. *rhodochrous* J1 for industrial applications.

## Materials and methods

### Strains, media, and culture conditions

*E*.*coli* JM109 was used for recombinant plasmid construction. *E*.*coli* BL21 (DE3) was used as the host for recombinant expression in this study. *E*. *coli* BL21 (DE3) transformants harboring recombinant plasmids were grown in 2×YT medium (16 g/L tryptone, 10 g/L yeast extract, 5 g/L NaCl) containing CoCl_2_·6H_2_O (1 g/L) and kanamycin (50 mg/L) at 37°C, and isopropyl β-D-thiogalactopyranoside (IPTG) was subsequently added to a final concentration of 0.6 mM. The cells were incubated at 200 rpm in the shaker at 24°C for 18 h.

*R*. *rhodochrous* J1 was from the stock in our laboratory, which has been used previously [8] and was used as a control in the present study. Pre-culture was performed at 30°C for 48 h in a 500 mL flask containing 50 mL of seed medium containing 0.3 g glucose, 0.3 g yeast extracts, 0.04 g urea, 0.016 g KH_2_PO_4_, 0.016 g K_2_HPO_4_, and 0.016 g MgSO_4_. Ten milliliters of the pre-culture was then inoculated into 100 mL fermentation medium of a 500 mL shaking flask containing 2.5 g glucose, 6 g yeast extract, 0.7 g urea, 0.06 g KH_2_PO_4_, 0.06 g K_2_HPO_4_, 0.06 g MgSO_4_, 0.1 g sodium glutamate, and 0.027 g CoCl_2_·6H_2_O. Incubation was performed at 28°C for 120 h [18].

### Construction of plasmids

**Fig 1:**
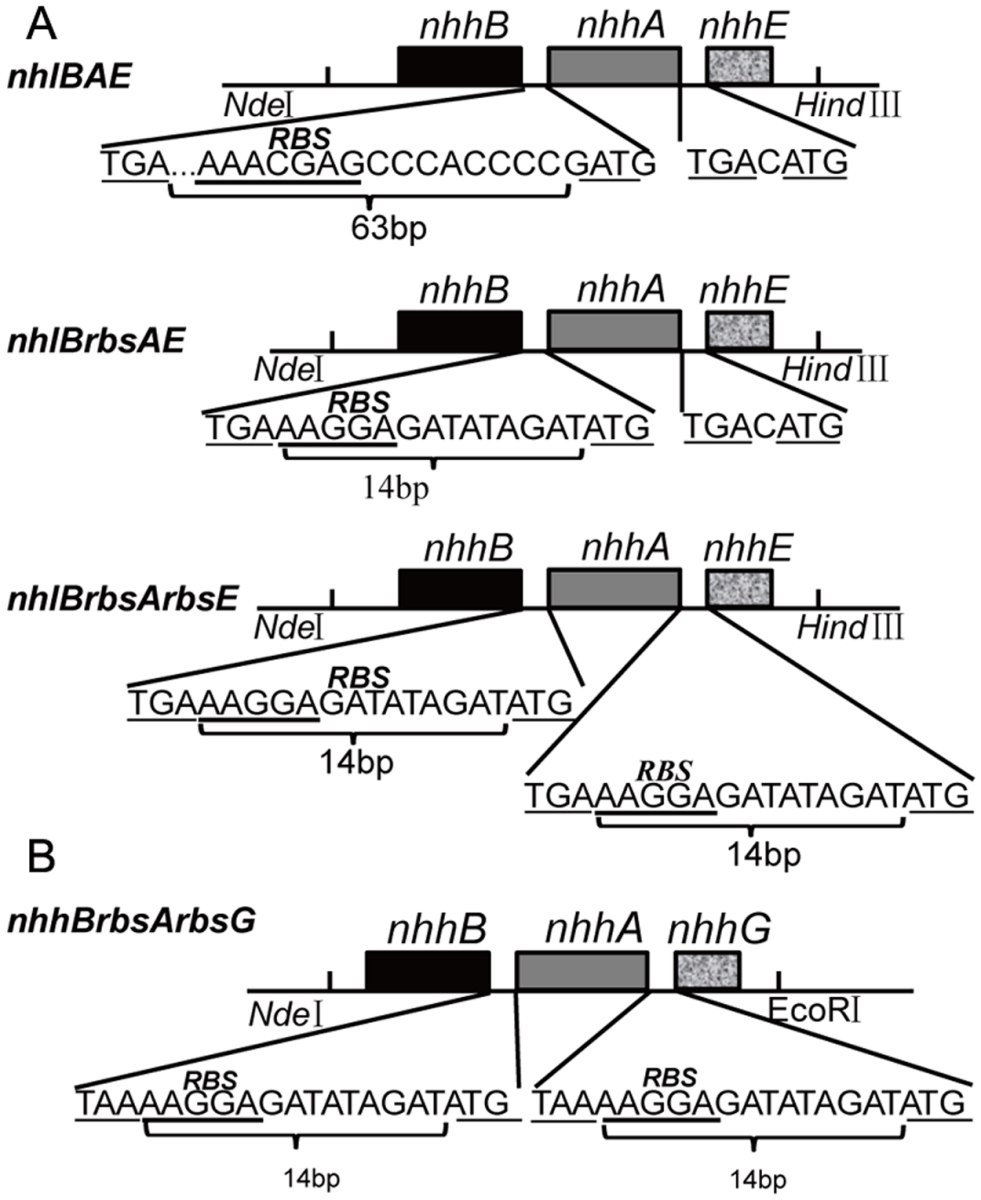
Gene organization for the construction of a set of plasmids. (A) Organization of the expression cassette for L-NHase. *nhlBAE*, wild-type L-NHase gene; *nhlBrbsAE*, the intervening sequences shortened gene with one enhanced RBS ahead of *nhlA; nhlBrbsArbsE*, the intervening sequences shortened gene with two enhanced RBSes ahead of *nhlA* and *nhlE*. (B) Gene organization of the constructed genes used for H-NHase expression.

The primers used in this study were listed in Table 1. The plasmid pREIT19-*nhlBAE* from *R*. *fascians* DSM43985 [8] was isolated and used as a template to clone the *nhlBAE* gene (GenBank: DJ492951.1) with the primer pairs B-up and E-down (Table 1). The PCR product and the vector pET24a(+) were simultaneously digested with *Nde*I-*Hind*III, and the two fragments were jointed together, generating pET24a-*nhlBAE* (Fig 1A). Plasmids pET24a-*nhlBrbsAE* and pET24a-*nhlBrbsArbsE*, which contained a strong RBS sequence in front of *nhlA* and *nhlE*, respectively, were constructed using pET24a-*nhlBAE* as a template. Reverse PCRs were performed with primer pairs 1RBS-up and 1RBS-down, 2RBS-up and 2RBS-down, respectively (Fig 1A). To facilitate the purification and separation, Strep-tag was fused into the plasmid pET24a-*nhlBrbsArbsE* in front of *nhlB* by reverse PCR with primer *Strep*-B-up and *Strep*-B-down, generating plasmid pET24a-*(B-Strep)rbsArbsE*.

**Table 1:** Primers used for NHase expression.

Name	Sequence(5’-3’)	Length (bp)
B-up	AAGGAGATATACATATGGATGGAATCCAC	29
E-down	CCCAAGCTTCTACCCGTCGGAGTCAGTGGTGC	32
1RBS-up	CGAACCGTATCTGCTACCGGCCTGA**AAGGAGATATAGA**TATGACCGCCCACAATCCCG	58
1RBS-down	CGGGATTGTGGGCGGTCAT**ATCTATATCTCCTT**TCAGGCCGGTAGCAGATACGGTTCG	58
2RBS-up	CACCACACCCAGCAAGGCCTGA**AAGGAGATATAGAT**ATGCCCCGACTCAACGAACAA	57
2RBS-down	TTGTTCGTTGAGTCGGGGCAT**ATCTATATCTCCTT**TCAGGCCTTGCTGGGTGTGGTG	57
*Strep* -B-up	GTTTAACTTTAAG**AAGGAGATATAGAT**ATGGCAAGCTGGAGCCACCCGCAGTTCGAAAAGGATGGAATCCACGACCTCGGTGGCC	85
*Strep* -B-down	GGCCACCGAGGTCGTGGATTCCATCCTTTTCGAACTGCGGGTGGCTCCAGCTTGCCATATGTATATCTCCTTCTTAAAGTTAAAC	85

The underlined sequences are the restriction sites. The bold and underlined sequences are the ribosome binding sites and spacer sequences.

The H-NHase genes were chemically synthesized with optimized codons based on the original H-NHase genes (GenBank: X86737.1) from *R*. *rhodochrous* J1, in which the strong RBS was inserted in front of *nhhA* and *nhhG*, resulting in the construction of *nhhBrbsArbsG*. The genes were inserted into pET24a to construct pET24a-*nhhBrbsArbsG*. The plasmid pET24a-*nhhBAG* harboring the native gene of H-NHase (*nhhBAG*) saved in our lab was used as a control for H-NHase expression in *E*. *coli*.

### Purification and identification of the recombinant proteins

All purification steps were performed at 4°C. All procedures were carried out by an AKTA purifier (GE Healthcare UK Ltd.). *E*. *coli* transformant containing the *Strep*-tagged L-NHase was collected after centrifugation. Cells were lysed by ultra-sonication after being washed three times with Strep Binding Buffer (SBB) composed of 20 mM Na_2_HPO_4_, 280 mM NaCl, 6 mM KCl, and 0.5 mM dithiothreitol (DTT), pH 7.4. Next, the cells were re-suspended in the same volume of SBB prior to ultra-sonication. The supernatant and precipitation were separated by centrifugation at 4°C for 10 min at 18,000×g. The supernatant was loaded onto an affinity column (*Strep*-tag/*Strep*-Tactin affinity chromatography, GE Healthcare UK Ltd, USA) equilibrated with SBB and eluted with Strep Elution Buffer (2.5 mM d-Desthiobiotin, SBB, 0.5 mM DTT, pH 7.4) [19].

The purification step of H-NHase was as described in a previously reported method with certain modifications [15]. Potassium Phosphate Buffer (KPB) (10 mM, pH 7.4) containing 0.5 mM DTT was used over the purification process according to the previous report[5]. Briefly, after ultra-sonication, H-NHase was partly purified by ammonium sulfate fractionation (40–65%). Afterward, the dialyzed solution was loaded onto the Hitrap Q HP (GE Healthcare) column for separation and purification by a linear gradient elution with the concentration of KCl from 0.2 to 0.6 M in KPB. The solutions were fractionated automatically. The purity of L-NHase and H-Hase were subsequently determined by SDS-PAGE analysis.

### Molecular mass determination of the purified proteins

Gel-filtration chromatograph analysis was performed for molecular mass determination, and MW-MARKER PROTEINS (ORIENTAL YEAST CO.,LTD) were used as a standard, which was in accordance with a previous report [8] with minor modifications. In this study, a Superdex 200 10/300 GL pg column (GE Healthcare UK Ltd.) equilibrated with 10 mM KPB (pH 7.4) was used for estimation of the molecular masses of the enzymes. This step was conducted with an AKTA purifier (GE Healthcare UK Ltd.) at 4°C, with a flow rate of 0.5 mL/min.

### Determination of protein concentration and metal content

The purified protein was analyzed with the absorbance value at 280 nm with B-500 ultramicro ultraviolet spectrophotometer (Yuan Analysis Instrument Co., Ltd. Shanghai, China). The metal content of the purified enzymes at a concentration of 0.5 mg/ml were analyzed with a Shimadzu AA-7000 atomic absorption spectrophotometer under the following conditions: wavelength, 240.73 nm; lamp current, 12 mA; and slit width, 0.2 nm.

### Enzymatic catalysis assay

The NHase activity was assayed as the procedures used previously [5]. The reaction mixture of a total volume of 0.5 mL was comprised of 10 mM KPB (pH 7.4), 400 mM 3-cyanopyridine as substrate, and 0.1 mg/ml purified enzyme. The reaction was performed at 25°C for 10 min and stopped by the addition of 0.5 mL acetonitrile. The amount of nicotinamide produced in the reaction mixture was determined as previously described [11]. One unit of NHase activity was defined as the amount of enzyme that catalyzed the production of 1 μmol of nicotinamide per min at 25°C. All of the experiments were performed independently in triplicate. The values were presented as mean±sd.

### Substrate and product tolerance determination

To determine the substrate tolerance of the enzymes, the reaction was carried out in a mixture of a total volume of 0.5 mL containing 0.1 mg/ml purified enzyme and 0.4, 0.8, 1.0 M of 3-cyanopyridine in 10 mM KPB (pH 7.4), respectively. The activities under different substrate concentration were compared, and the activity using 0.4 M of 3-cyanopyridine was defined as 100%. For the product tolerance determination, 0.5 mL of mixture containing 0.1 mg/ml purified enzyme and 0.5, 1.0, 1.5 M of nicotinamide in 10 mM KPB (pH 7.4) were treated for half an hour at 25°C. Next, 0.1 M of 3-cyanopyridine was added into the mixture for reaction. The decrement of 3-cyanopyridine was measured by HPLC in the same method as nicotinamide and used for assay, and the activity without nicotinamide treatment was defined as 100%. To assay the nicotinamide-tolerance of NHases in recombinant *E*. *coli* and wild-type *R*. *rhodochrous*, we performed the measurement according to the method in a previous report with minor modification [20]. We prepared cells in 0.5 M nicotinamide for 20 min at 25°C. Next, 3-cyanopyridine was added prior to the enzymatic assay. The activity without the addition of a high concentration amide was defined as 100%. The relative activity was the ratio of which the activity of treated NHases accounted for the untreated NHases.

## Results and discussion

### Overexpression of L-NHase in *E*. *coli*

**Fig 2:**
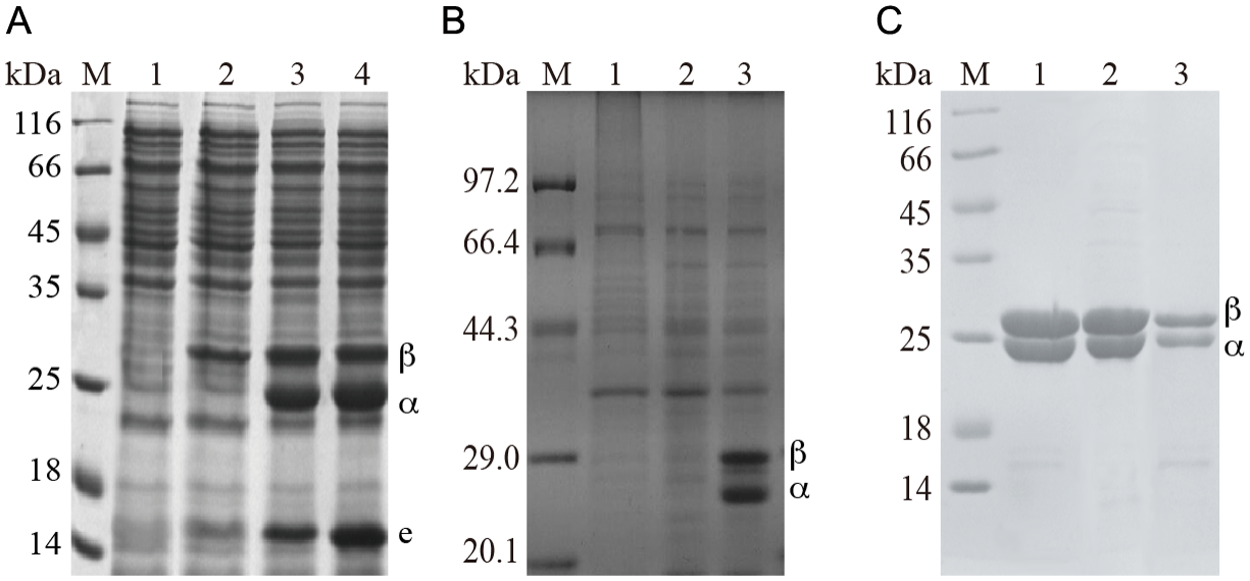
SDS-PAGE analysis of the expression of L-NHases and H-NHase. (A) The expression of L-NHases. M: Marker; 1: control, BL21(DE3)/pET24a; 2: BL21(DE3)/pET24a-*nhlBAE*; 3: BL21(DE3)/pET24a-*nhlBrbsAE*; 4: BL21(DE3)/pET24a-*nhlBrbsArbsE*. (B) The expression of H-NHase. M: Marker; 1: control, BL21(DE3)/pET24a; 2: BL21(DE3)/pET24a-*nhhBAG*; 3: BL21(DE3)/pET24a-*nhhBrbsArbsG*. (C) The purified L-NHases and H-NHase. M: Marker; 1, L-NHase encoded by *nhlBrbsAE*; 2, L-NHase encoded by *nhlBrbsArbsE*; 3, H-NHase encoded by *nhhBrbsArbsG*.

To heterologously express L-NHase in *E*. *coli*, the gene cluster of *nhlBAE* (including β and α-subunit-encoding genes and the self-subunit swapping chaperone-related gene *nhlE*) were inserted into pET24a resulting in the recombinant plasmid pET24a-*nhlBAE*. *E*. *coli* transformant harboring pET24a-*nhlBAE* was used for expression of L-NHase. As shown in Fig 2A, lane 2, the band of the β-subunit of L-NHase was clearly seen on SDS-PAGE, while the bands of the α-subunit and NhlE were scarcely observed on the gel. This finding indicates that the routine method for over-expression of *nhlBAE* in *E*. *coli* is essentially adaptively engineered to augment the quantity of the recombinant protein. It has been shown that heterologous expression of proteins in *E*. *coli* can be enhanced by employing a synthetically strong ribosome binding site (RBS) [21] and by reducing the distance between the promoter region and target gene [22]. Therefore, strategies involving substitution of strong RBS and space deletion were executed. The most preferred sequence of strong RBS in *E*. *coli* is believed to be AAGGA [23]. To enhance the expression of the α-subunit and NhlE, the putative RBS (AAACGAG) between the *nhlB* and *nhlA* genes (Fig 1A) was replaced with an enhanced RBS (AAGGA), and the spacing sequences between the two genes were shortened from 63 bp to 14 bp, constructing plasmid pET24a-*nhlBrbsAE*. However, NhlE is essential for the activity of L-NHase. To enhance the expression of NhlE, the strong RBS (AAGGA) was also inserted between *nhlA* and *nhlE*, yielding the expression plasmid pET24a-*nhlBrbsArbsE* (Fig 1A). *E*. *coli* transformants harboring pET24a-*nhlBrbsAE* and pET24a-*nhlBrbsArbsE* were used for L-NHase expression. As shown in Fig 2A (lane 3 and lane 4), the bands of the α- and β-subunits and NhlE of L-NHase were clearly detected on SDS-PAGE, and the activity in cell-free extracts of the transformants harboring pET24a-*nhlBrbsAE* and pET24a-*nhlBrbsArbsE* were 120 U/mg and 132 U/mg, respectively.

Almost all of the natural mRNAs have RBS upstream of the start codon, which functions at the step of translation initiation. The efficiency for binding of ribosome to the RBS is determined by the complementarity between RBS and the ribosome [24]. The putative RBS sequences of the native α-subunit and the NhlE are AAACGA, which is a weak RBS for recombinant expression in *E*. *coli*. The strategy involving substitution of an enhanced RBS (AAGGA) may help the heterologous expression of L-NHase in *E*. *coli*. In addition, the spacer between the start codon AUG and RBS, which plays an important role in regulation of translation initiation efficiency, affects the incorporation of fMet-tRNA into the ribosome [25]. In the present study, we reduced the spacer between the start codon AUG and RBS of mRNA of the α-subunit from 63-bp to a 9-bp, which may be another factor enhancing L-NHase expression. These engineering strategies resulted in the successful expression of L-NHase in *E*. *coli*.

### Overexpression of the type of high-molecular-mass NHase (H-NHase) in *E*. *coli*

In addition to the strategies involving substitution of strong RBS and reducing the distance between the promoter and target gene, heterologous expression of proteins in *E*. *coli* can also be enhanced by gene codon optimization [26]. With the aforementioned success in overproducing L-NHase, as well as gene codon optimization, we attempted to express H-NHase in *E*. *coli*. The gene *nhhBAG* with the optimized codon (including the β and α-subunit and the self-subunit swapping chaperone *nhhG* genes) were designed and chemically synthesized, in which the putative RBS upstream of the *nhhA* gene was replaced by an enhanced RBS (AAGGA). In addition, NhhG is essential for H-NHase activity. To augment the expression of NhhG, the enhanced RBS was also inserted between *nhhA* and *nhhG*. The synthesized genes were digested and ligated into pET24a, constructing plasmid pET24a-*nhhBrbsArbsG* (Fig 1B). *E*. *coli* transformant harboring pET24a-*nhhBrbsArbsG* was used for H-NHase expression. As a control, *E*. *coli* transformant harboring pET24a-*nhhBAG* (the native H-NHase genes) was also used for H-NHase expression. As a result, the bonds corresponding to the α- and β-subunits of H-NHase were obvious on SDS-PAGE of strain harboring pET24a-*nhhBrbsArbsG* (Fig 2B), and the activity in the cell-free extracts was 85 U/mg, while bonds corresponding to the α- and β-subunits of H-NHase was undetectable on SDS-PAGE of strain harboring the native H-NHase genes.

### Characterizations of the recombinant L-NHase and H-NHase

**Fig 3:**
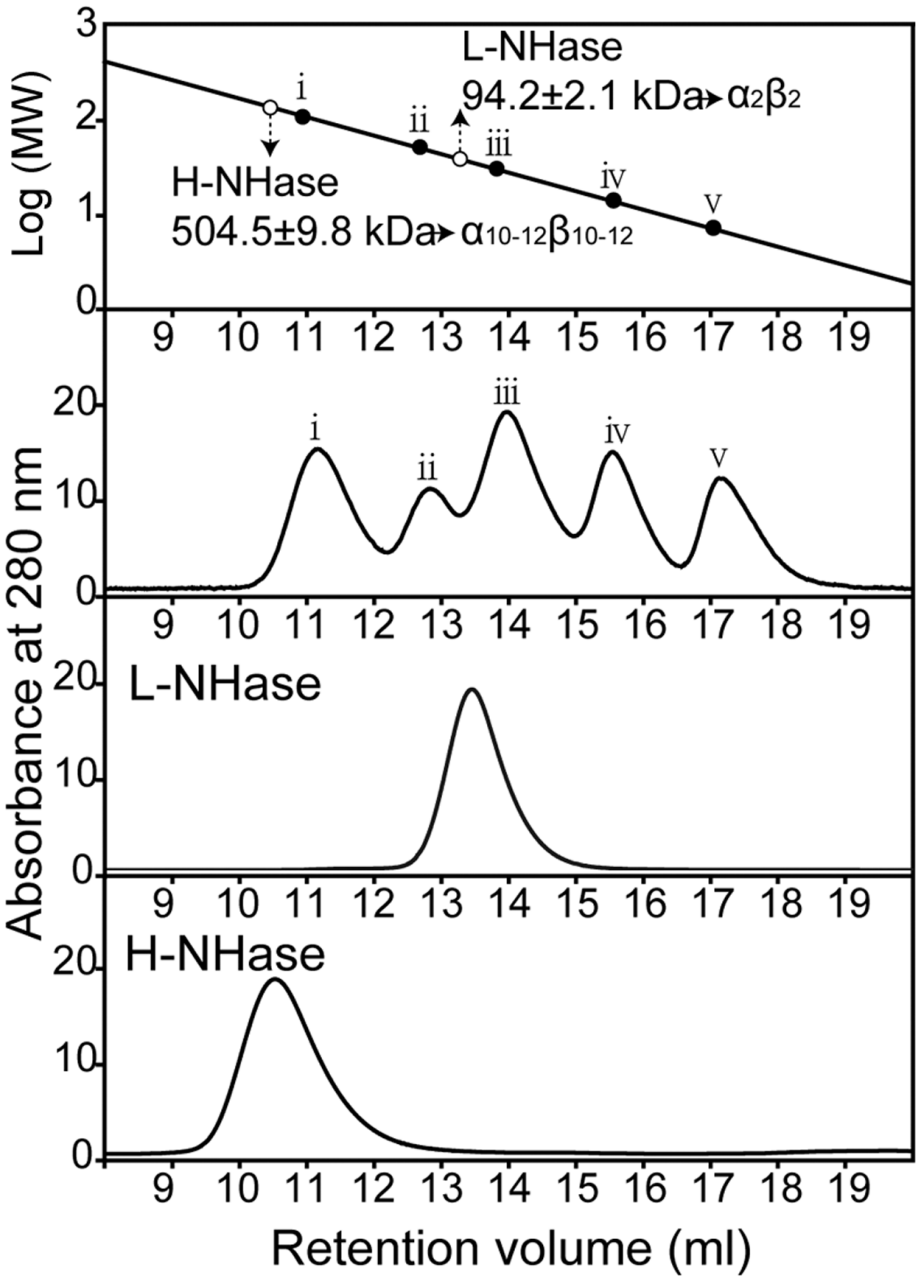
Determination of the molecular masses of purified proteins. Determination of the molecular masses and structures of NhlBA and NhhBA by Superdex 200 10/300 GL pg column. The marker proteins were used for gel filtration: (i) glutamate dehydrogenase (yeast) (290 kDa), (ii) lactate dehydrogenase (pig heart) (142 kDa), (iii) enolase (yeast) (67 kDa), (iv) myokinase (yeast) (32 kDa), and (v) cytochrome c (horse heart) (12.4 kDa).

The L-NHases expressed from the transformants harboring pET24a-*nhlBrbsAE* and pET24a-*nhlBrbsArbsE*, as well as H-NHase expressed from the transformant harboring pET24a-*nhhBrbsArbsG*, were purified (Fig 2C) and characterized. The activities are shown in Table 2, and the specific activities of the purified L-NHases were 390 U/mg and 430 U/mg, respectively, which were similar to that from the wild type and expressed in *R*. *facians* DSM43985 [8]. The specific activity of the purified H-NHase was 235 U/mg, which was the same level as that from the wild type and that expressed in *R*. *rhodochrous* ATCC12674 [15]. The cobalt contents of these NHases were 0.8, 0.9, and 0.8 mol/mol of αβ, indicating that these enzymes were cobalt-containing NHases and that the cobalt incorporation successfully occurred in the *E*. *coli* recombinant system. The molecular masses of the recombinant L-NHase and H-NHase were analyzed by gel filtration. As shown in Fig 3, the molecular mass of the L-NHase was determined to be 94 kDa. As the calculated molecular masses of α and β-subunits of L-NHase are 22.8 kDa and 25.2 kDa, respectively, the recombinant L-NHase comprised of α_2_β_2_ should be 96.0 kDa, which was the same as the wild-type L-NHase. The molecular mass of the H-NHase was determined to be 504 kDa. Because the calculated molecular masses of the α- and β-subunits of H-NHase are 22.8 kDa and 26.3 kDa, respectively, the recombinant H-NHase comprised of α_10_β_10_ should be 490 kDa, which corresponds to that of the wild-type H-NHase (α_10–12_β_10–12_) [18]. These findings suggest that both L-NHase and H-NHase from *R*. *rhodochrous* J1 are actively expressed in *E*. *coli*, and the recombinant enzymes retain the native forms and properties.

**Table 2:** Characterization of purified NHases.

Protein	cobalt content (mol of irons/mol of protein)	specific activity (units/mg)
L-NHase (nhlBrbsAE)	0.8±0.1(per αβ)	390±9.5
L-NHase (nhlBrbsAE)	0.9±0.1(per αβ)	430±12.1
H-NHase	0.8±0.1(per αg)	235±7.6

The values represent the means±sd for quadruplicate independent experiments. The corresponding proteins were the same concentration: 0.5 mg/mL for determination of cobalt ion incorporation, 0.1 mg/mL for specific assay.

The activity of L-NHase encoded by the expression cassette *nhlBrbsArbsE* was slightly higher than that by *nhlBrbsAE*, which might be due to the higher expression level of NhlE in the cassette organization of *nhlBrbsArbsE* than that of *nhlBrbsAE*. NhlE acts as a self-subunit swapping chaperone for cobalt incorporation into L-NHase. NhlE functions in a dimer with the α-subunit of L-NHase, forming αe_2_. The incorporation of cobalt to L-NHase depends on the exchange of the α-subunit between the cobalt-free α-subunit of the L-NHase and the cobalt-containing αe_2_ [8]. The higher expression level of NhlE increases the formation of αe_2_, resulting in a more complete α-subunit exchange between the cobalt-free L-NHase and cobalt-containing αe_2_. This result could be supported by the cobalt content in L-NHases encoded by *nhlBrbsArbsE* and *nhlBrbsAE* (0.9, 0.8 mol/mol of αβ, respectively).

### Comparison of thermos-stability, substrate and product tolerance of L-NHase and H-NHase

**Fig 4:**
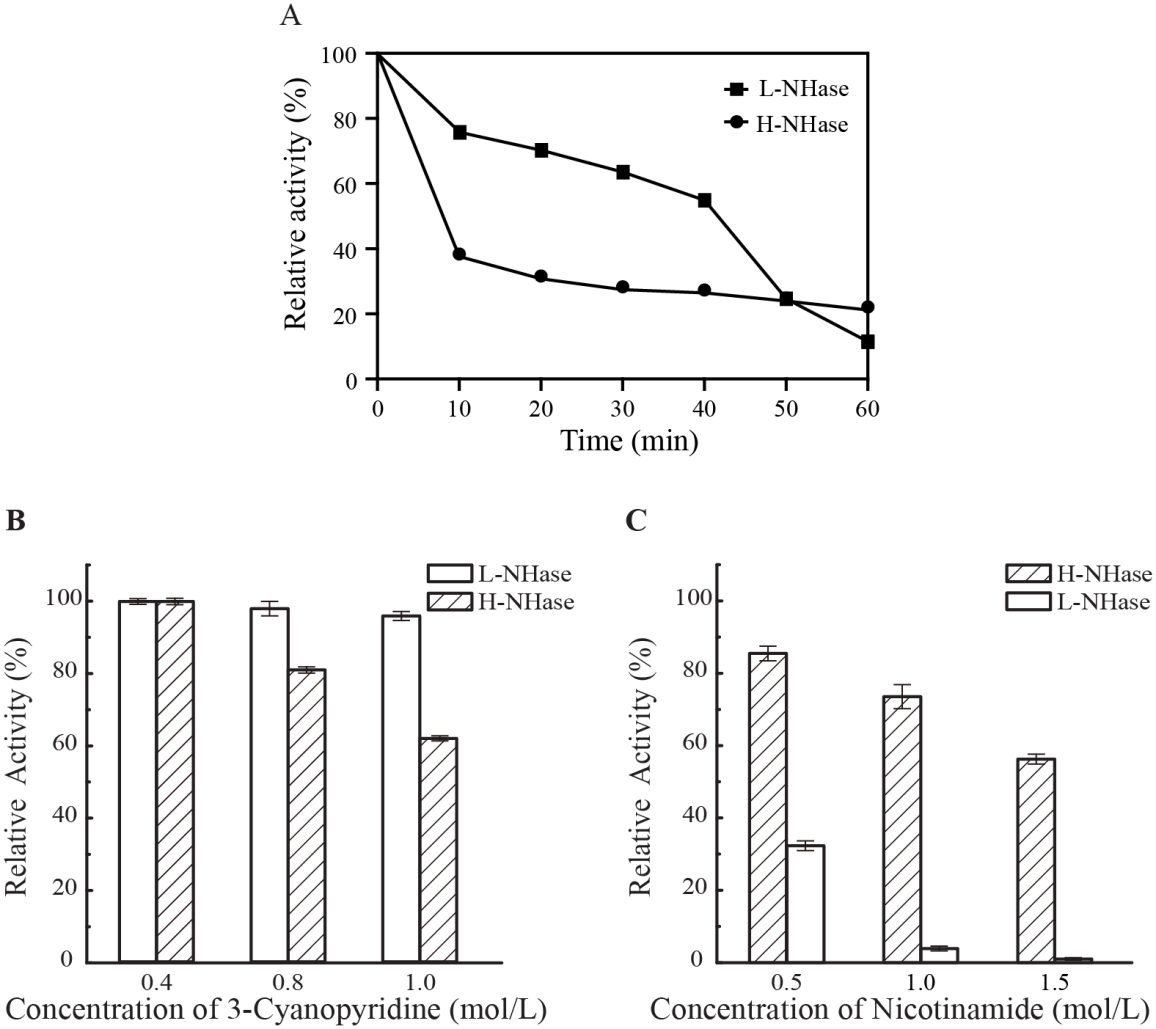
Thermo-stability, substrate and product tolerance of L-NHase and H-NHase. (A) Thermo-stability of NHases; (B) Substrate tolerance of enzymes; (C) Product tolerance of enzymes.

Thermos-stability, substrate and product tolerance of an enzyme are the important factors for the enzyme application. To determine the thermos-stability, we incubated the purified enzymes at 50°C for 60 minutes and measured their activity every ten minutes. As shown in Fig 4A, L-NHase exhibited higher thermos-stability than H-NHase. However, substrate and product tolerance of H-NHase was significantly better than those of L-NHase (Fig 4B and 4C). The activity of L-NHase decreased 40% under the condition of 1 M substrate (3-cyanopyridine), while almost no inhibition could be observed for the H-NHase in the same condition (Fig 4B). The activity of L-NHase was reduced sharply after treatment by 0.5 M product (nicotinamide), in contrast, H-NHase still has 60% of activity after treatment by 1.5 M nicotinamide. H-NHase may be more suitable for industrial application because of its higher product tolerance, which would lead to a high product concentration.

### Production of NHase by recombinant *E*. *coli* is superior to that in *R*. *rhodochrous* J1

**Fig 5:**
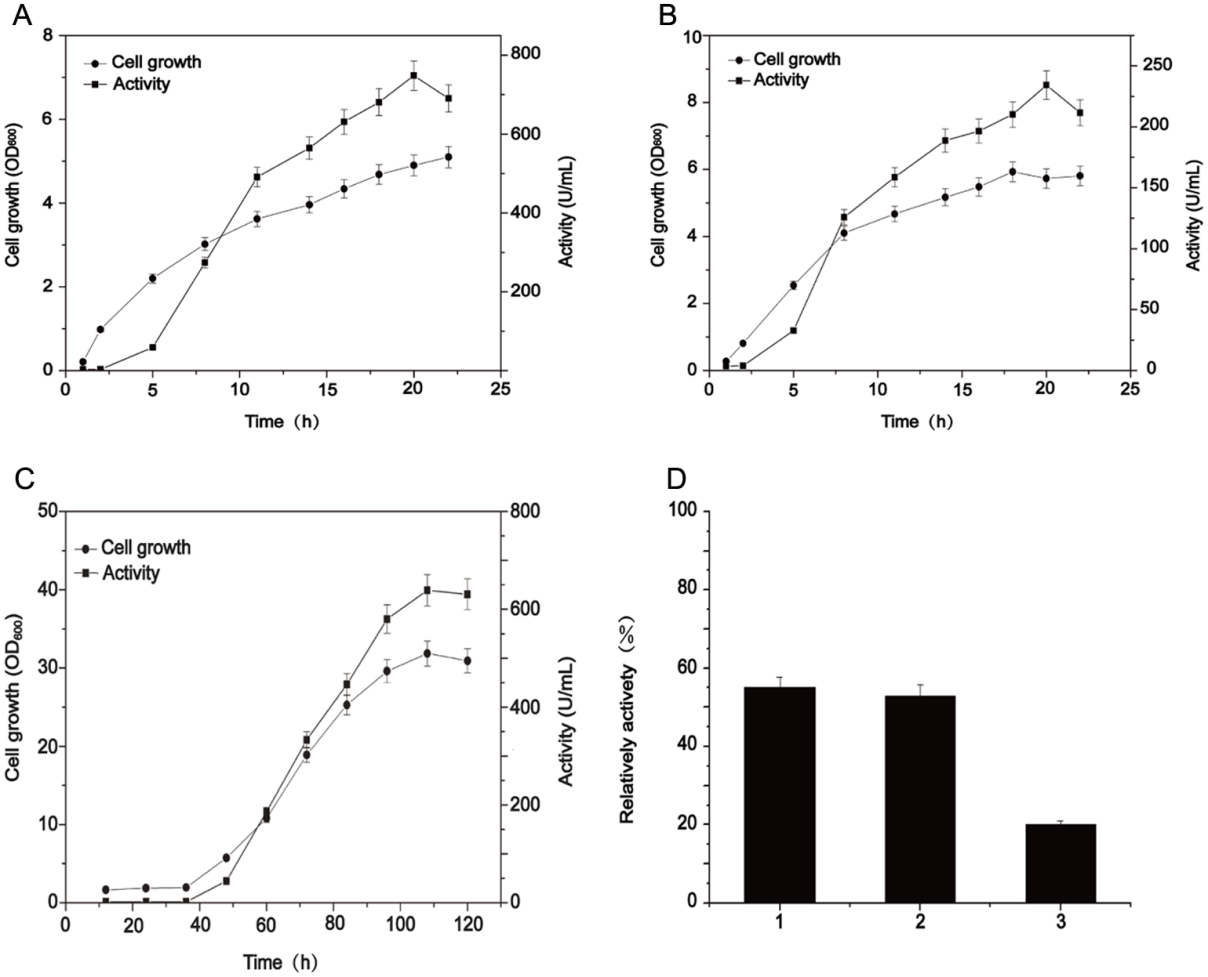
Production of NHase in recombinant *E*. *coli* and in native *R*. *rhodochrous* J1. Cell growth and NHase activity of (A) the genetically engineered *E*. *coli* for H-NHase, (B) L-NHase, and (C) *R*. *rhodochrous* J1. (D) Comparison of nicotinamide-tolerance of NHases from the cells of *R*. *rhodochrous* J1 (column 1) and the H-NHase, and L-NHase from the genetically engineered *E*. *coli* (column 2 and 3). The reduction ratio (the proportion of the reduced 3-cyanopyridine amount in the reaction with and without 0.5 M nicotinamide) was calculated.

The culture condition and catalytic properties for overproduction of NHase were compared between recombinant *E*. *coli* and native *R*. *rhodochrous* J1 to highlight which host is superior to produce NHase. As shown in Fig 5, the cell growth of the transformants harboring pET24a-*nhlBrbsArbsE* (for L-NHase) and pET24a-*nhhBrbsArbsG* (for H-NHase) were faster than *R*. *rhodochrous* J1, and the activities of L-NHase and H-NHase peaked at 20-h cultivation, which was 750 U/ml and 240 U/ml, respectively (Fig 5A and 5B). Regarding the *R*. *rhodochrous* J1, the highest total NHase activity was 650 U/ml, which was achieved after 110-h cultivation (excluding the seed culture time for *R*. *rhodochrous* J1) (Fig 5C). In parallel, the values of OD_600_ of *E*. *coli* transformants harboring L-NHase and H-NHase genes when reaching maximum enzyme activities were 5.0 and 5.4, respectively, while the OD_600_ of *R*. *rhodochrous* J1 was 30 (Fig 5C). The activity unit per cell of the two recombinant *E*. *colis* overproducing L-NHase and H-NHase were 150, 44.4 U/OD_600_, respectively, which were much higher than that of *R*. *rhodochrous* J1(21.7 U/OD_600_). To compare the product tolerance of NHases in recombinant *E*. *coli* and in wild-type *R*. *rhodochrous*, we tested the tolerance to nicotinamide in these strains. The reactions were carried out in mixtures containing 400 mM 3-cyanopyridine (substrate) with and without 0.5 M nicotinamide (product) for 20 min. The decrease of 3-cyanopyridine in each reaction was measured, and the reduction ratio (the proportion of the reduced 3-cyanopyridine amount in the reaction with and without 0.5 M nicotinamide) was calculated (Fig 5D). The reduction ratios of L-NHase and H-NHase in genetically engineered *E*. *coli* were 20% and 53%, respectively. The reduction ratio for the wild-type strain was 55%, indicating that the H-NHase recombinant *E*. *coli* exhibit similar product tolerance to that of the wild-type strain.

Regardless of the immobilized cells or free NHase, the enzyme should be exposed to an environment with a high concentration of product at the late phase during biotransformation. Tolerance to a high concentration of product is an essential property that NHase must possess for industrial applications. Although the product-tolerance for L-NHase-harboring recombinant *E*. *coli* was slightly lower than that for *R*. *rhodochrous* J1, the H-NHase-harboring recombinant *E*. *coli* displayed equivalent product-tolerance compared to that of *R*. *rhodochrous* J1. The low activity in the culture of recombinant *E*. *coli* harboring H-NHase could be eliminated by augmenting the quantity of biomass through high-density fermentation. In addition, the capacity for product-tolerance of NHase could be enhanced by protein engineering [27]. The best known strategy is to fuse self-assembling peptide to the N-terminus of NHase, which can largely enhance the capacity for tolerance to high-concentration product [27]. This strategy may also elevate the tolerance to product in recombinant *E*. *coli* harboring L-NHase.

## Conclusions

**Fig 6:**
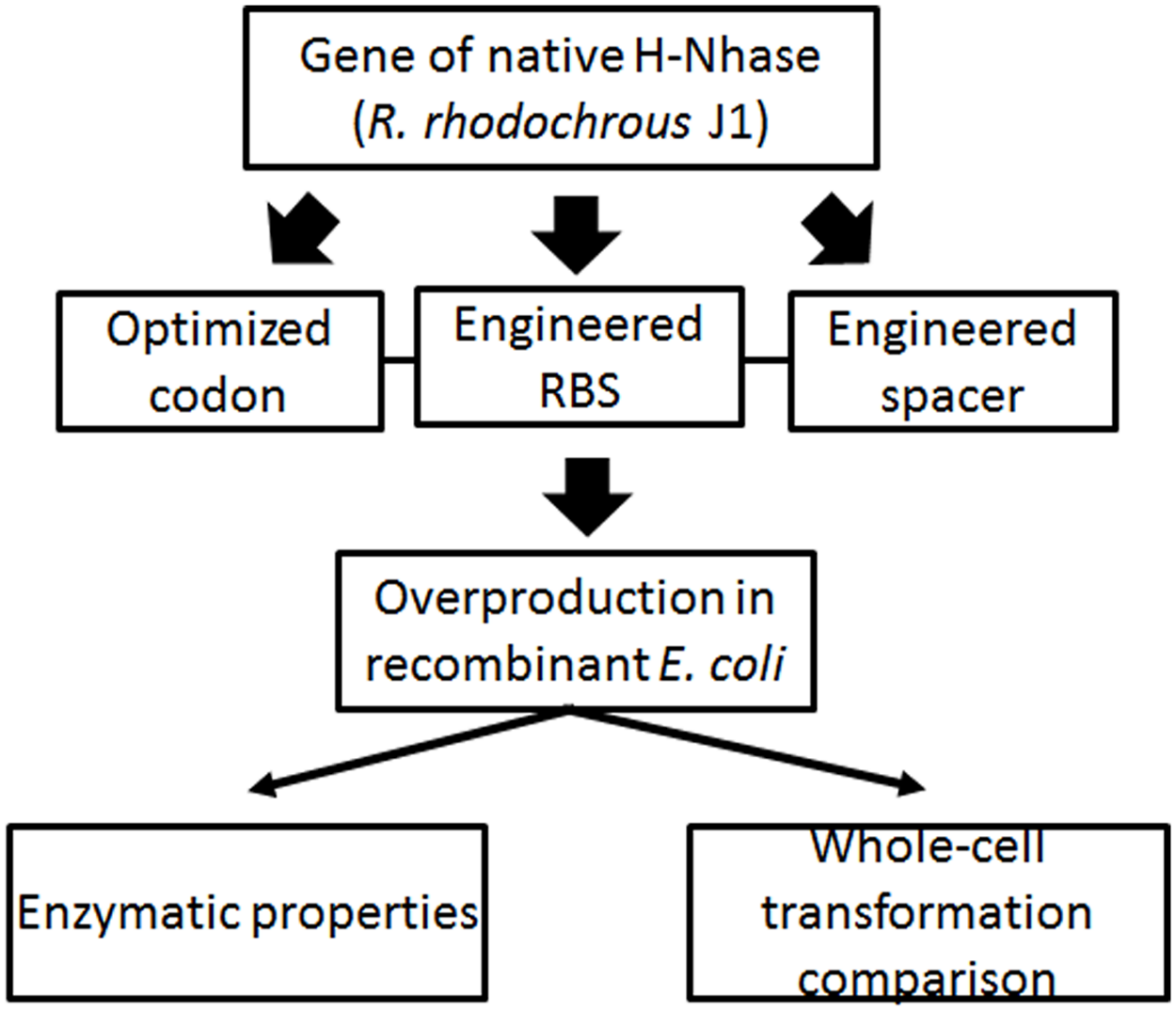
The flow chart to illustrate the strategy for this study. The sketch depicted the overall framework for this study. Genetic engineering of native NHase was carried out in two tiers. Firstly, the codons were optimized. Secondly, the genetic elements, RBS and spacer, were modified and optimized. Resorting to these modifications, the optimized NHases were over-produced in recombinant E. coli. Accordingly, the enzymatic properties were characterized and a transformation pipeline by whole-cell catalysis was primarily established.

The two NHases from *R*. *rhodochrous* J1 were overexpressed in *E*.*coli* via codon-optimization, substitution of stronger RBS, and optimization of spacing sequences between different genes. Production of NHase by recombinant *E*. *coli* is superior to that in *R*. *rhodochrous J1*. It is possible that the genetically engineered *E*. *coli* could be used for industrial applications instead of *R*. *rhodochrous* J1 (Fig 6).
